# In vitro synergism of fosfomycin and clarithromycin antimicrobials against methicillin-resistant S*taphylococcus pseudintermedius*

**DOI:** 10.1186/1471-2180-14-129

**Published:** 2014-05-19

**Authors:** Matthew DiCicco, Suresh Neethirajan, J Scott Weese, Ameet Singh

**Affiliations:** 1 BioNano Laboratory, School of Engineering, University of Guelph, Guelph N1G 2 W1, Canada; 2 Department of Pathobiology, Ontario Veterinary College, University of Guelph, Guelph N1G 2 W1, Canada; 3 Department of Clinical Studies, Ontario Veterinary College, University of Guelph, Guelph, Guelph N1G 2 W1, Canada

**Keywords:** Staphylococcus, Biofilms, Clarithromycin, Fosfomycin, Antimicrobials, Synergistic effect, MRSP

## Abstract

**Background:**

Bacterial biofilms are of tremendous concern for clinicians, as they can compromise the ability of the immune system and antimicrobial therapy to resolve chronic and recurrent infections. Novel antimicrobial therapies or combinations targeted against biofilm establishment and growth subsequently represent a promising new option for the treatment of chronic infectious diseases. In this study, we treated bacterial biofilms produced by methicillin-resistant *Staphylococcus pseudintermedius* (MRSP) with a combination of fosfomycin and clarithromycin. We selected these agents, because they prevent biofilm formation and induce antimicrobial synergism that may also target other staphylococci.

**Results:**

We determined that the combination of fosfomycin and clarithromycin better impairs *S. pseudintermedius* biofilm formation compared to treatment with either therapy alone (P < 0.05). Morphological examination of these biofilms via scanning electron microscopy demonstrated that fosfomycin alone does impact biofilm formation on orthopaedic implants. However, this activity is enhanced in the presence of clarithromycin. We propose that the bacteriostatic activity of clarithromycin is accentuated when fosfoymcin is present, as it may allow better penetration into the biofilm matrix, allowing fosfomycin access to sessile bacteria near the surface of attachment.

**Conclusions:**

Here, we demonstrate that the combination of fosfomycin and clarithromycin may be a useful therapy that could improve the clinical outcomes of treating antimicrobial resistant MRSP biofilms.

## Background

The extensive use of antimicrobials during the last half century has promoted the evolution of antimicrobial resistance characteristics in pathogenic and opportunistic microorganisms [1,2]. The selective pressures induced by antimicrobial therapies have forced the acquisition and spread of a variety of antimicrobial resistance determinants. Resistance mutations may arise spontaneously or certain organisms may derive these from foreign DNA encountered at sites of infection. Many organisms have steadily gained resistance due to their ability to uptake DNA from the surrounding environment and incorporate it into their genome. For example, Falsetta [3] studied N. *gonorrhoeae*, which is naturally competent and gains resistance by using several systems of DNA uptake to acquire foreign DNA. At the same time, several strains actively release their DNA into the environment. Thus, resistance genes can come from self-organisms and non-self-organisms. In addition to the development of resistance, many pathogenic and opportunistic bacterial species utilize other strategies that enable them to evade clearance from their host, such as of the formation of biofilm structures that are recalcitrant to removal [4]. Although the definition of a biofilm has fluctuated over the last 20 years, classically biofilms are defined as microorganisms that are irreversibly attached to a surface, which are encased in a protective (often self-produced) matrix that may be composed of eDNA, exopolysaccharides, host material, shed membranes, etc. [5,6]. These organisms tend to work cooperatively to ensure community survival, where some may forfeit active growth [7,8]. As a result, biofilm infections are generally difficult to clear, owing to enhanced microbial resistance and tight adherence to the surface of the substrate [6,9]. There are several theories as to why bacterial biofilms are so resistant to antimicrobial therapy, which may exist in tandem with one another: i) the matrix impedes the penetration of antimicrobials into the biofilm, ii) many cells within the biofilm are not metabolically active and are thus resistance to many antimicrobials therapies, iii) biofilms are actively resistant through the acquisition of resistance genes and/or the expression of efflux pumps, and iv) biofilms contain a subpopulation of cells that are not susceptible to antimicrobials (e.g. resistors) [4,9]. As a result, the minimum inhibitory concentration (MIC) of biofilm-embedded bacteria can be 10 to 1000 times higher than their planktonic counterparts, which often represents a dose that would be lethal to the host [10,11].

Due to the potential impact of biofilms on the development and persistence of serious and life-threatening infections and the difficulty in eliminating them, understanding the mechanisms used to produce them in clinically relevant bacteria along with the identification of potentially novel strategies to prevent or remove them is paramount. *Staphylococcus pseudintermedius* is a critically important, opportunistic, canine pathogen found in skin, soft tissue, and surgical site infections (SSIs) [12]. Methicillin-resistant strains (MRSP) are of concern, because of their inherent resistance and ability to form biofilms [13,14]. Overall, MRSP may be a good model of methicillin resistant biofilms that may have application to human methicillin resistant infections [15]. In vitro studies of other staphylococcal strains have shown that biofilm-associated SSIs may be reduced through combinational antimicrobial therapy [16]. Clarithromycin (CLA), a semi-synthetic broad spectrum macrolide, has fairly potent *in vitro* and *in vivo* anti-biofilm activity against Gram-positive *S. aureus* alone and in combination with other antimicrobials, independent of its antimicrobial activity [16-18]. A recent study indicated that clarithromycin alone had little to no effect on biofilm formation by MRSP [19], yet a combinational therapy remained to be evaluated. Therefore, we elected to test such a therapy on MRSP biofilms.

Fosfomycin (FOS) has been reported to destroy biofilm and increase penetration of other antimicrobials into the biofilms of both Gram-positive and Gram-negative bacteria [20-22]. This antimicrobial has been shown to interfere with the synthesis of peptidoglycan in the cell wall and enters susceptible bacteria by means to two different transport uptake systems: the L-α-glycerophosphate transport system (GlpT) and the hexose–phosphate uptake system (UhpT) [23]. Fosfomycin provides adequate distribution into tissues in clinically relevant concentrations, and it has been suggested that its high degree of tissue and biofilm penetration is attributed to its low molecular weight and negligible protein binding [24]. However, despite these favourable pharmacokinetic properties and notable effects against bacterial biofilms, the emergence of resistance can preclude its use as a single agent.

The use of combination antimicrobial regimens with FOS could help to reduce the risk of antimicrobial resistance as well as provide a synergistic effect with other antimicrobials including beta-lactams, aminoglycosides, and fluoroquinolones [22,25,26]. Interestingly, synergistic studies have demonstrated that FOS may even decrease the level of penicillin-resistance in pneumococci by altering the degree of expression of penicillin-binding proteins [27]. When used in combination, FOS appears to exert substantial antimicrobial activity and may be clinically effective against infections caused specifically by “problem” Gram-positive cocci pathogens both *in vitro* and *in vivo *[28,29]. In support to this, we found that FOS in combination with CLA is highly effective in reducing biofilm biomass in vitro, more so than either therapy alone. We suggest that this may be an effective therapy to reduce biofilm-related wound infections. Further study is warranted to test its impact in vivo; this study lays the foundation for that work.

## Results and discussion

Structurally unrelated to other antimicrobials, FOS uniquely inhibits the first step of peptidoglycan biosynthesis in bacterial cell wall by binding to UDP-*N*-acetyl-glucosamine enolpyruvate transferase [23]. Its low molecular weight (194.1 Da) and non-reactivity with the negatively charged bacterial glycocalyx allows for efficient diffusion into tissues and the biofilm matrix [30]. This may explain its enhanced antimicrobial activity against biofilm embedded bacteria, as it has been shown to destabilize biofilms and thereby enhance the permeability of other antimicrobials [20,22,31].

### Fosfomycin and clarithromycin synergistic activity

**Figure 1:**
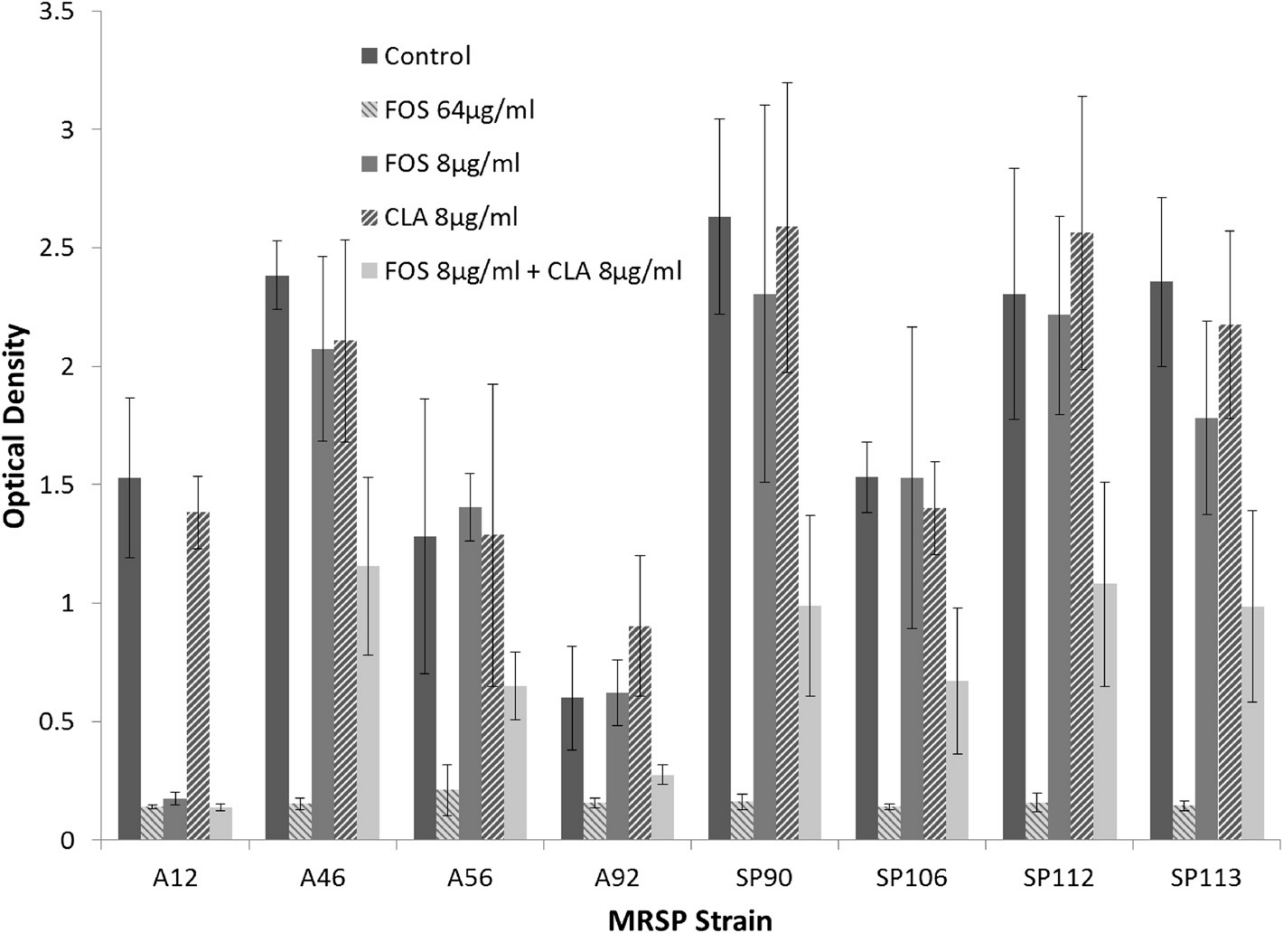
**Enhanced antibacterial activity of fosfomycin (FOS) and clarithromycin (CLA) against MRSP following 24 h growth.** Biofilm forming potential of one ST68 strain (A12) and seven ST71 strains (A46, A56, A92, SP90, SP106, SP112, SP113) and the effect of FOS and CLA in mono and combination therapy. Combination therapy had a significant effect (P < 0.05) while low doses of FOS and CLA alone had no significant effect (P > 0.05) on MRSP biofilm formation.

Microtitre plate assay (MPA) results identified synergism between CLA and FOS in reducing biofilm production. Fractional inhibitory concentration index (FICI) values (Table 1) revealed fractional synergy (FICI ≤ 0.5) of 0.31 to 0.56 in the FOS and CLA resistant strains. As a set 1:1 combination of FOS and CLA (Breakpoint dose for CLA resistance is ≥ 8 μg/ml) was chosen, the FIC may be lower based on specific MIC against biofilm for each strain. In comparison with the control samples, low doses of FOS at 8 μg/ml (P > 0.05) and CLA at 8 μg/ml (P > 0.05) independently produced no significant reduction in biofilm production, whereas treatment with FOS and CLA in combination resulted in a significant (P < 0.05) reduction in the bacterial biomass (Figure 1) in one-way ANOVA models. To ensure that this impact was directed against biofilm formation and was not simply inhibiting bacterial growth both FOS resistant (≥64 μg/ml) and CLA resistant (≥256 μg/ml) strains were chosen. As the strains tested are resistant to FOS in high doses, and MRSP has been found to resist the anti-biofilm effect of CLA in mono-therapy [19], this demonstrated synergism between FOS and CLA in an *in vitro* setting is particularly interesting.

**Table 1:** Interaction of fosfomycin and clarithromycin against MRSP biofilms by microdilution arrays

**Isolate selected**	**Sequence type**	***Dru *** **type**	**Adherence capabilities**	**FOS (μg/ml) MIC**	**CLA (μg/ml) MIC**	**FICI**
**A12**	68	10h	STRONG	≥1	≥256	NA
**A46**	71	9a	MODERATE	≥64	≥256	0.31
**A56**	71	9a	LOW	≥32	≥256	0.56
**A92**	71	9a	MODERATE	≥64	≥256	0.31
**SP90**	71	9a	STRONG	≥32	≥256	0.56
**SP106**	71	9a	LOW	≥64	≥256	0.31
**SP112**	71	9a	LOW	≥64	≥256	0.31
**SP113**	71	9a	LOW	≥64	≥256	0.31

Adherence capabilities were determined based on the model developed by Stepanovic *et al*., 2000. Fosfomycin and Clarithromycin susceptibility was determined by agar dilution and Kirby Bauer disk diffusion, respectively.

### Potential mechanism of synergism against MRSP

The mechanism behind the synergism between the fosfomycin and clarithromycin is unknown. In *S. aureus*, cellular adhesion is mediated by adhesive matrix molecules which are covalently anchored to the cell wall peptidoglycan [32,33]. In addition, extracellular matrix fibronectin can serve as a bridging molecule between several bacterial species and variety of host type cells or non-biological surfaces [34]. *S. pseudintermedius* expresses surface proteins that resemble those from *S. aureus* and has the capacity to bind to the fibrinogen, fibronectin, and cytokeratin of host cells [35]. Cell wall associated adhesive proteins, particularly the fibrinogen-binding protein ClfA present on the surface of *Staphylococcus pseudintermedius*, is a candidate therapeutic target for the control of bacterial pyoderma on skin infections [35]. It also produces an immunoglobulin-binding protein called staphylococcal protein A (Spa), similar to that of S. *aureus *[34]. Although speculative, FOS may alter these binding mechanisms through its interference with peptidoglycan biosynthesis of the bacteria.

Quorum sensing regulates biofilm formation and cell-cell communication in bacteria, and it can be influenced by the combined antimicrobials against MRSP biofilms [36,37]. The accessory gene regulator (agr) quorum sensing and signal transduction has been described in *S. aureus *[38,39], which mediates bacterial oxidation response via intramolecular disulfide redox switch, which was also very recently identified in *S. pseudintermedius *[40]. Quorum sensing in Gram-positive bacteria has been found to regulate a number of physiological activities, including induction of virulence factors in *S. aureus*. Macrolide antimicrobials have been shown to affect quorum sensing within biofilms, leading to reduced polysaccharide synthesis and instability of the biofilm architecture [41,42]. Thus, it is possible that FOS may also influence the quorum-sensing signals of these strains. We plan to investigate this further in future studies by examining mRNA expression of agr and or protein levels in response to FOS treatment.

### Surface coverage and morphological effects of fosfomycin

**Figure 2:**
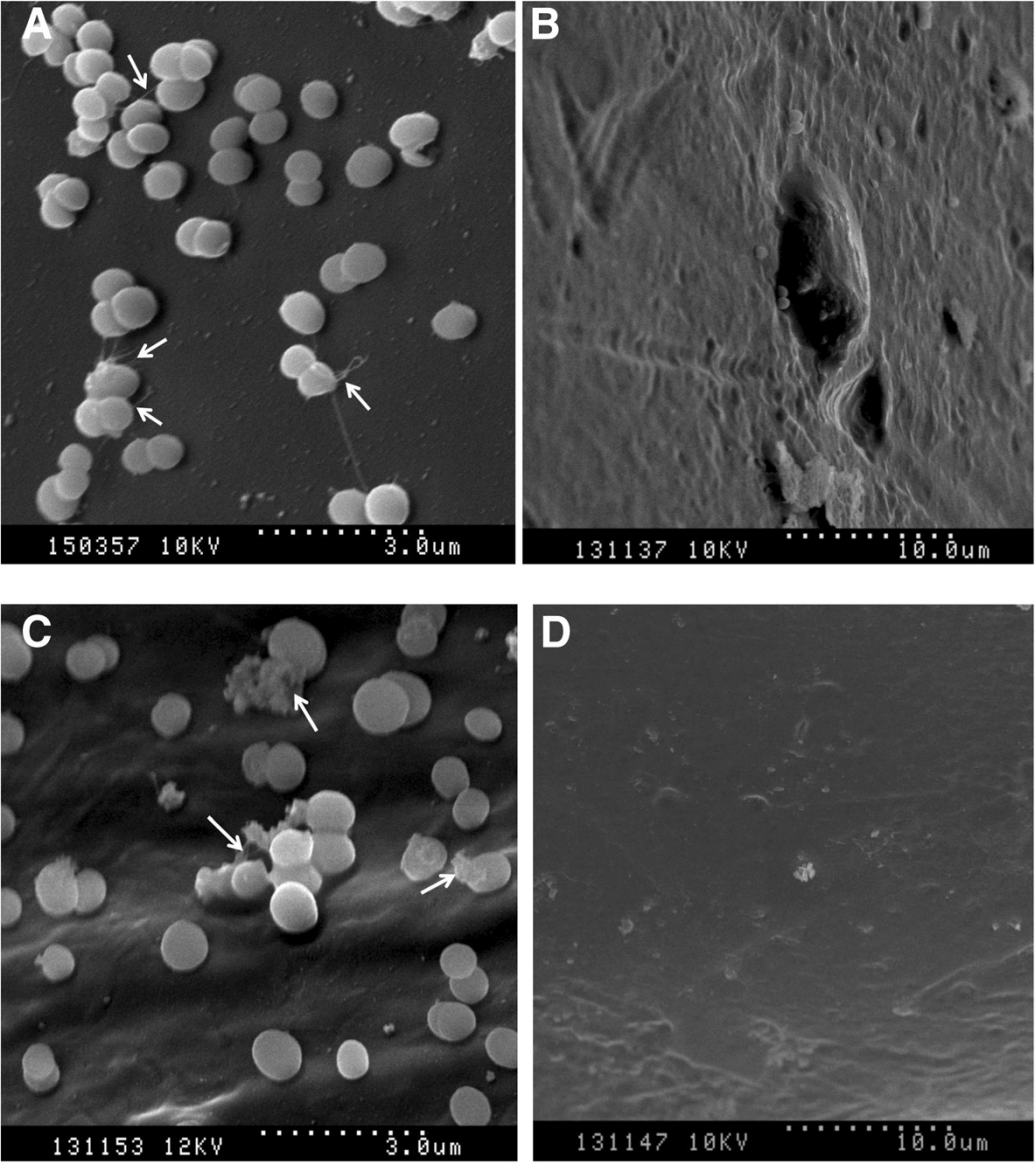
**Characteristic cell morphologies of MRSP biofilms and its surface coverage on titanium orthopaedic screws.** The effect of fosfomycin against MRSP A12 strain on titanium orthopaedic screws was assessed microscopically. Scanning electron micrographs of 4 and 24 h old MRSP biofilms on orthopaedic screws are shown without **(A)**, **(C)** and treated with fosfomycin **(B)**, **(D)** respectively. The biofilm cells embedded in biofilm extracellular matrix is indicated by the arrows in the control samples.

**Figure 3:**
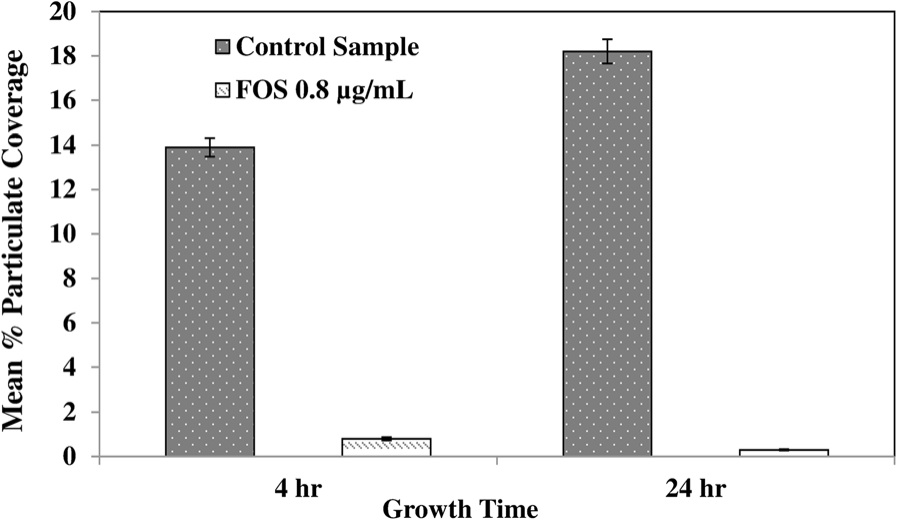
**Percent biofilm coverage on orthopaedic screw surface over 4 and 24 h time periods.** Image analysis of particulate coverage of SEM images demonstrates that a significant difference (P < 0.05) exists between treated and untreated samples. Extracellular polymeric substances and adherent and biofilm-embedded cells were highlighted against the background in the same locations across both samples.

Monotherapy with concentrations of FOS below the selected strain’s MIC were also found to reduce adherence and biofilm structure on titanium orthopaedic screws. The percent particulate (clusters of biofilms) on the orthopaedic screw surfaces decreased significantly (P < 0.05) between control and FOS treated samples. In control samples, complicated fibrous structures, biofilm-embedded cells, and colonies of bacteria were noted as early as 4 h with increasing amounts of surface coverage after 24 h of growth (Figure 2A and C). Comparisons between the samples indicated that surface area coverage by MRSP biofilm decreased from 13.9% to 0.8% due to FOS treatment over 4 h and from 18.2% to 0.3% over 24 h (Figure 3). A decreased change in extracellular polymeric substance production and the density of adherent bacteria and biofilm structures was also noted at 4 h in samples treated with 0.8 μg/ml of FOS (Figure 2A and B). There is a significant difference in biofilm coverage between the control and FOS treated samples; biofilm coverage is reduced by treatment, indicating higher efficacy and the potential for preventing MRSP adhesion on clinically relevant surfaces. Further, enumeration (Table 2) of biofilm collected from titanium screws confirmed that FOS (at below-MIC levels) significantly decreased biofilm formation (P < 0.05).

**Table 2:** Average number of MRSP bacterial colonies grown from titanium screws treated with and without fosfomycin (n = 3)

**Dilution factor**	**Average number of bacterial colonies (CFU)**	
	**Control**	**0.8 μg/ml FOS**
**1:10**^**-1**^	468 ± 16.7	4.6 ± 0.5
**1:10**^**-2**^	47.2 ± 1.5	0
**1:10**^**-3**^	4.2 ± 0.4	0
**1:10**^**-4**^	0	0

The values represent the mean and standard deviation of 3 replicates from two independent experiments.

**Figure 4:**
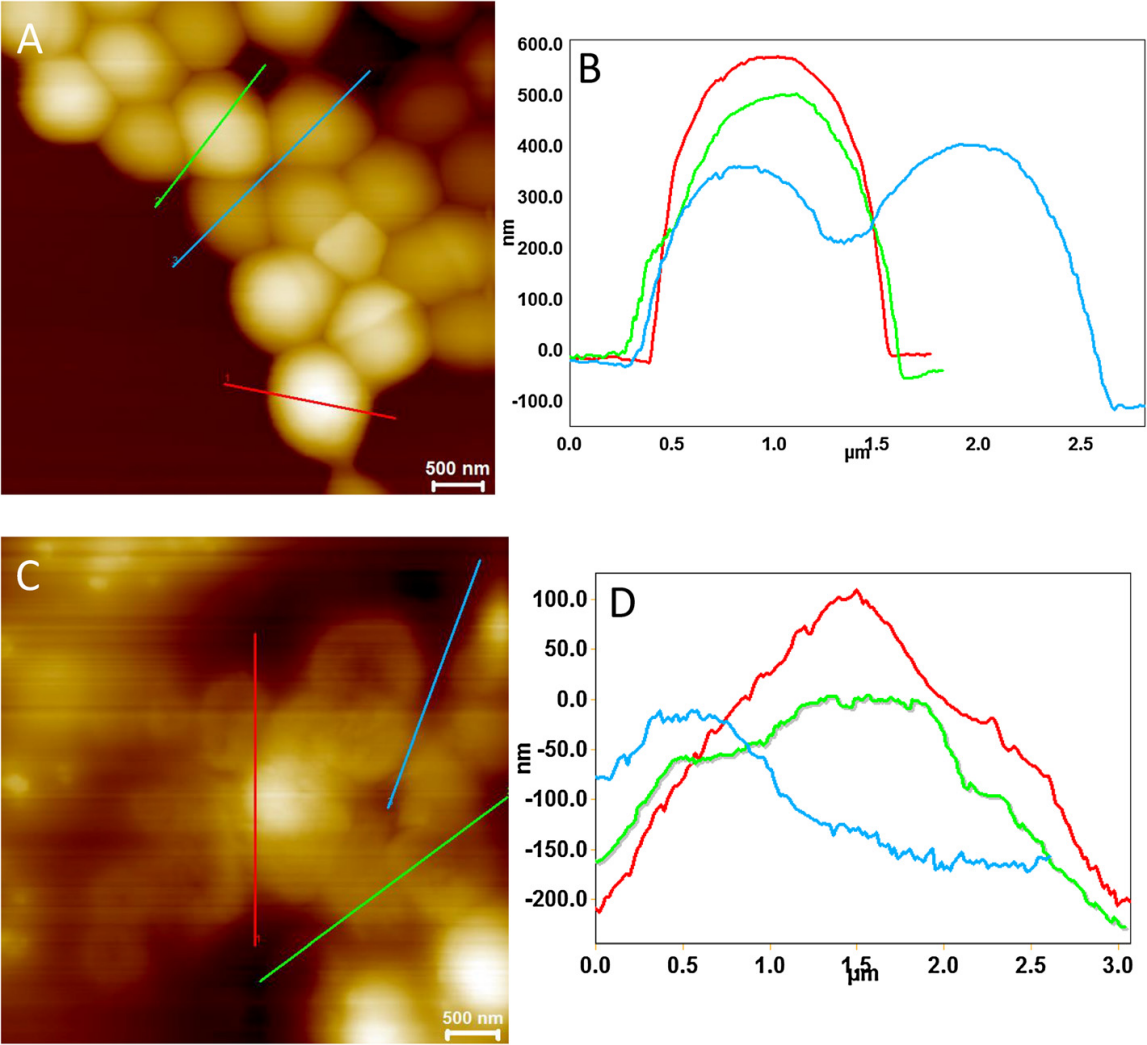
**MRSP biofilm surface height profiles with corresponding AFM deflection mode images (Scale = 5 μm). (A)**, **(B)** MRSP A12 AFM image showing clusters of biofilms with extended chains exhibiting stable nanoscale morphology. **(C)**, **(D)** Fosfomycin treated MRSP biofilms for 4 h exhibits greater deviation in nanoscale morphology and reduced height indicating the efficacy of fosfomycin. The cellular ultrastructure has been significantly altered with less surface coverage and a smaller cell diameter.

Assessment of the effect of FOS on MRSP biofilm through AFM revealed distinct morphological variations when comparing large clusters of cocci shaped biofilms in untreated controls and treated samples (Figure 4). The cocci shape is evident in the control sample, while the cells appear to have lysed in the FOS treated samples. The cellular morphology was dramatically altered and the cells appeared to be collapsed, which is indicative of lysis following FOS treatment. Untreated (control) MRSP biofilms grown over 4 h on mica sheets had a significantly larger diameter (1 μm) compared to the FOS-treated MRSP biofilms, which were an average of 97 nm in diameter. In the treated samples, MRSP cells were well dispersed and isolated, appearing to be damaged with a greatly lowered height. The AFM image analysis clearly indicates that the effect of FOS on MRSP was significantly detrimental, indicating the possibility of cell-wall degradation. SEM and AFM image analysis data agree with the MPA data and provide further evidence of fosfomycin’s effect against MRSP growth *in vitro*.

### Combination therapy benefits

Synergistic approaches have been shown to reduce the possibility of resistance gaining in systemic therapy and have been proven effective in reducing this occurrence for *Pseudomonas aeruginosa* and *Escherichia coli* in both *in vitro* testing and *in vivo* trials [43,44]. In addition, development of cross-resistance to FOS through the use of other antimicrobial agents has been regarded as insignificant, likely due to its unique bioactivity against bacteria [45,46]. For these reasons the use of FOS/CLA in combination therapy may prove effective for MRSP biofilm-forming strains in a clinical setting to reduce recurrent SSIs on indwelling biomaterials. However, additional in vivo and *in vitro* studies using biofilm models across larger populations of strains and *in vivo* studies are warranted.

As an in vitro study, this study is focused on using clinical isolates that are naturally resistant in a biofilm model being more representative than planktonic growth. The obtained results will serve the agenda of investigating the polymicrobial wound infection models, and will aid in predicting the response in the complex natural environment of the biofilm.

It is unclear whether the synergistic effect on MRSP biofilm noted here would also apply to other staphylococcal species, and study of the effect of this combination of other clinically relevant staphylococci is needed. This study also only investigated MRSP, not methicillin-susceptible *S. pseudintermedius* (MSSP). It is reasonable to extrapolate results to MSSP given the lack of evidence of an association between methicillin-resistance and either biofilm production or resistance to fosfomycin.

## Conclusions

Results show that FOS and CLA in combination have a significant effect on biofilm formation *in vitro*, independent of their antimicrobial activity and in contrast to monotherapy results. A synergistic effect between FOS and CLA was noted that increased the apparent the effectiveness of FOS and CLA, despite the fact that the strains tested were determined to be resistant to either therapy alone. *In vivo* and further *in vitro* trials evaluating the effect of these two antimicrobials in combination on simulated 3D wound infection models are warranted. Our results indicate that a combinational therapy of FOS and CLA may be highly effective in preventing biofilm formation by MRSP strains, even those predisposed to resistance to either agent alone. Therefore, this therapy may be promising in the treatment of resistant biofilm wound infections. Our next steps will be to investigate a simulated wound infection model in microfluidic systems, to test other strains isolated from dogs, and further characterize the effect of the therapy on biofilm structure using methods that hydrate or distort the biofilm, such as confocal microscopy. In the end, we could foresee using the combination of FOS and CLA as preventative agents either in a topical application or as an oral dose to limit the potential for MRSP biofilm formation. Alternatively, we intend to test their ability to disrupt already established biofilms as a therapeutic agent once biofilm infection has been identified. These agents may be more successful than the currently available modalities, as they are effective together at doses that could be safely administered to patients without obvious negative impact. These agents are already used clinically alone, so they are ideal agents for a combination therapy and would be both safe and effective.

## Methods

### Ethics statement

Bacterial isolates from dogs were collected as part of studies that were approved by the University of Guelph Animal Care Committee.

### Bacterial isolate screening

We tested 31 epidemiologically unrelated MRSP isolates from dogs from Canada and the United States were screened for biofilm production via microtiter plate assay (MPA) [47,48], FOS and CLA resistance by agar dilution and Kirby Bauer disk diffusion [49,50] respectively, and further characterized by sequence analysis of the *mec*-associated direct repeat unit (*dru* typing) [51]. Clinical and Laboratory Standards Institute (CLSI) approved susceptibility breakpoints do not exist for FOS against staphylococci, and instead European Committee on Antimicrobial Susceptibility Testing (EUCAST) breakpoint was used to determine susceptibility with an MIC ≤ 32 μg/ml [14,52]. For susceptibility testing, 25 μg/ml glucose 6-phosphate (G6P) was added to the agar plates to improve FOS uptake [23,53,54].

### Evaluation of biofilm production

To determine biofilm adherence characteristics, strains were first cultured aerobically for 24 h at 35°C in Columbia Agar with 5% sheep blood before suspension at a 0.5 McFarland standard (~10^8^ CFU/ml) in tryptic soy broth supplemented with 1% glucose (TSB-G) + 25 μg/ml G6P. We transferred 200 μl of each inoculum to a 96-well polystyrene microtiter plate in triplicate and incubated aerobically for 24 h at 35°C. This was followed by washing of the wells with phosphate buffered saline (PBS) three times to remove non-adherent cells, and heat fixation at 60°C for 1 h. Crystal violet 0.1% (w/v) was then applied for 15 minutes to dye the cells before drying at room temperature overnight, and resolubilization of adherent cells with 95% ethanol. Used as an indication of biofilm production, optical density (OD) measurements were taken of the wells at 570 nm (OD_570_), and were averaged over each strain and subtracted from the readings of the negative control (wells containing uninoculated media). Strains were classified as biofilm producers if OD_570_ was >0.200 and further classified as weak (0.600 > OD_570_ ≥ 0.200), moderate (1.200 > OD_570_ ≥ 0.600) and strong (OD_570_ ≥ 1.200) biofilm formers [48].

### Impact of FOS and CLA on biofilm production

To assess potential synergism against biofilm formation, independent of antimicrobial activity, seven biofilm producing (OD_570_ > 0.200) MRSP isolates that were resistant to CLA and FOS were studied. The impacts of FOS, CLA, and FOS + CLA on biofilm formation were evaluated by microtitre plate assay (MPA) by comparing biofilm production with and without the antimicrobial therapy as described above. The selected isolates were treated with the following therapy: no treatment, high FOS (64 μg/ml), low FOS (8 μg/ml), CLA (8 μg/ml), and FOS (8 μg/ml) + CLA (8 μg/ml). Breakpoint doses for CLA resistance (≥8 μg/ml) [50] were chosen to represent a concentration that can be readily achieved *in vivo (i.e., safe and effective)*[42]. Antimicrobial synergy was assessed by the fractional inhibitory concentration index (FICI), represented by the following formula [43,55].

$$\mathrm{FICI}=\frac{\mathrm{MIC}\left(\mathrm{FOS}+\mathrm{CLA}\right)}{\mathrm{MIC}\left(\mathrm{FOS}\right)}+\frac{\mathrm{MIC}\left(\mathrm{FOS}+\mathrm{CLA}\right)}{\mathrm{MIC}\left(\mathrm{CLA}\right)}$$

FICI values were interpreted as synergistic (FICI ≤ 0.5), synergistic to additive (0.5 < FICI ≤ 1), indifferent (1 < FICI ≤ 4), and antagonistic (FICI > 4) [43].

### Scanning electron microscopy (SEM)

To assess the effect of FOS on MRSP adhesion to a different abiotic and clinically relevant surface, SEM was used to image bacterial adherence and the biofilm matrix on 316 LVM titanium 20 mm orthopaedic bone screws (Veterinary Orthopaedic Implants, St. Augustine, FL, USA). One strong biofilm producing MRSP isolate was chosen from the population and inoculated at a 0.5 McFarland standard suspension in 5 ml of TSB-G + 25 μg/ml G6P. The screws were added to test tubes containing the bacterial suspension with and without 0.8 μg/ml FOS—the MIC for the strain—and incubated at 35°C. At 4 and 24 h of incubation the screws were washed with PBS, fixed with 2.5% glutaraldehyde for 24 h and rinsed in Sorensen’s phosphate buffer for 15 min three times. This was followed by post-fixation in 1% osmium tetraoxide for 30 min at room temperature, washing in Sorensen’s phosphate buffer for 15 min two times, dehydration through an ethanol gradient (50-100%), critical-point drying, and finally sputter coating with gold. Samples treated with and without 0.8 μg/ml FOS were imaged at 4 levels (3, 10, 30, and 100 μm) at two locations —along the head and between the threads of the orthopaedic screws—using a Hitachi S-570 scanning electron microscope. Image acquisition location was standardized across all replicates in relation to the detector beam, with images taken in the top-right quadrant of the screw head, and second screw thread along the minor diameter. Percent particulate coverage of the surface of titanium orthopaedic screws was determined from multiple SEM images of the same region of interest using ImageJ image analysis program (National Institute of Health, Bethesda, USA). The gray-scale SEM images were converted to binary format and the percent white-to-black pixels were calculated for each of the images. The SEM images were also visually ranked for microbial biofilm morphology.

### Enumeration of biofilm on screws

Enumeration of adherent biofilm grown on titanium screws was completed after removal by sonication. The same high biofilm-forming strain from the population was grown over night before inoculation at a 0.5 McFarland standard suspension in 5 ml of TSB-G + 25 μg/ml G6P. Titanium screws were added to the inoculated media with and without 0.8 μg/ml of FOS and incubated for 24 h. Following incubation, the screws were removed from the inoculum, washed to remove non-adherent bacteria and then transferred to tubes containing fresh TSB-G. Samples were then sonicated for 2 min (Branson Ultrasonic Cleaner Model 2510, Emerson Industrial Automation, Danbury, USA) and vortexed for 15 s to disperse previously adhered biofilm amongst the media. Serial dilutions of 10^-1^ through 10^-5^ for each screw were plated and colony forming units (CFU) counted (n = 3) after overnight growth.

### Atomic Force Microscopy (AFM)

For morphological studies, one strong biofilm producing isolate as determined from the MPA study was chosen from the population and inoculated at a 0.5 McFarland standard suspension in 10 ml of TSB-G + 25 μg/ml G6P and grown to late mid-log phase. The cells in a 1 ml sub-sample were centrifuged in a Scilogex Model D3024 microfuge at 5000 g for 3 min at room temperature, and washed 3 times with sterile analytical-grade water. The pellet was again suspended in deionized distilled water and the concentration of the bacteria was measured by a spectrophotometer at 540 nm. Freshly cleaved mica sheets were added to petri-dishes containing the bacterial cell culture suspension with and without 0.8 μg/ml FOS and incubated for 4 h and 24 h at 35°C. Upon incubation, the mica sheets were gently removed using fine tip tweezers, washed in free-flowing nano-pure water to remove the freely attached cells and dried at room temperature for 3 hours before imaging. AFM imaging was carried out for both the control samples and the bacterial culture treated with FOS (n = 3). Analysis was done with duplicate cultures for each time point with cells imaged in air with a tapping mode atomic force microscope (Dimension Icon SPM, Bruker). AFM height, amplitude, and phase images were obtained in AC mode on the air-dried mica substrates. A triangular Si cantilever tip (Bruker AFM Probes, Camarilla, CA) with a spring constant of 0.35 N/m and a resonance frequency of 18 kHz was used. A scan speed of 0.7-1.5 Hz was set and resulted in a final resolution of 512 by 512 pixels.

### Statistical methods

Data from the MPA was analyzed through one-way ANOVA with post-hoc Tukey’s Range test to compare different treatments with the control with a P < 0.05 being considered significant. Mean particulate coverage on SEM images in two different areas of the screws were assessed with Kruskal–Wallis one-way ANOVA (P < 0.05). Enumeration profiles of biofilm adhered to screws was analyzed using Student’s t-test to compare biofilm growth between FOS treatment and the control (P < 0.05). All statistical analysis was performed on commercially available software (SAS 9.2 TS Level 2 M3; SAS Institute Inc., N.C., U.S.A).

## Competing interests

The authors declare that they have no competing interests.

## Authors’ contributions

MD designed experiments, and carried out micro-titre plate assays, SEM imaging and determined MIC assays, and prepared and drafted the manuscript. SN, and SW conceived the study. SN, SW and AM participated in the design and implementation and reviewed the manuscript. All authors read and approved the final manuscript.
